# Financial challenges and economic impact on families of children with autism spectrum disorder in Kazakhstan

**DOI:** 10.3389/fpubh.2026.1846395

**Published:** 2026-08-12

**Authors:** Gaukhar Kayupova, Aigul Muzafarova, Nailya DeLellis, Karina Nukeshtayeva, Marina Lyubchenko

**Affiliations:** 1 Institute of Life Sciences, Karaganda Medical University, Karaganda, Kazakhstan; 2 Department of Health Sciences, Central Michigan University, Mount Pleasant, MI, United States

**Keywords:** autism spectrum disorder, costs, financial burden, Kazakhstani case, support

## Abstract

**Background:**

Families of children diagnosed with autism spectrum disorder (ASD) often face challenges associated with treatment, caregiving, and education, which lead to substantial expenses. In Kazakhstan, the economic impact of ASD on families remains insufficiently studied. Therefore, our aim was to examine the financial burden and identify factors associated with financial difficulties among these families.

**Methods:**

A cross-sectional study was conducted at the Regional Children’s Psychiatric Dispensary in the Karaganda region of Kazakhstan in 2023. The survey was administered to parents of children with ASD under 18 years of age both in person and online. Of the 285 questionnaires distributed, 239 were included in the analysis. The primary outcome was the presence of financial difficulties among these families. Regression analysis identified several variables associated with monthly expenditures related to caring for a child with ASD.

**Results:**

Among the participating families, 90.79% reported experiencing financial difficulties related to caring for a child with ASD. Non-medical expenditures accounted for the largest proportion of total monthly expenditures (38.08%). Multivariable logistic regression showed that older child age was associated with lower odds of financial difficulties (OR = 0.76, *p* < 0.001), whereas families living in urban areas had higher odds of reporting financial difficulties than those living in rural areas (OR = 6.30, *p* < 0.05).

**Conclusion:**

These findings provide insights into the economic challenges experienced by families of children with ASD in Kazakhstan and suggest the importance of continued development of government support.

## 1 Introduction

Autism spectrum disorder (ASD) is a neurodevelopmental condition affecting communication, cognition, and intellectual functioning, and is often associated with behavioral issues such as repetitive actions (1). Globally, ASD prevalence is estimated at 62 per 10,000 individuals (2). The number of registered children with ASD in Kazakhstan increased from 77 in 2003 to 3,000 in 2017 (3).

As the number of children with ASD grows, so does the demand for social support and specialized services, creating a substantial financial burden for families (4). For example, the lifetime cost per individual with autism in the United States is estimated at USD 3.2 million (5). Within the United Kingdom, the lifetime cost of ASD has been estimated based on functional level and associated costs (6). In Scotland, annual ASD-related costs were estimated at nearly USD 3.08 billion (7). In Ireland, out-of-pocket expenses averaged USD 9,576.79 per child annually, while public spending was approximately USD 16,604, of which around USD 15,019 was related to education (8). In Australia, the estimated national cost of ASD ranges from USD 3.1 billion to USD 4.9 billion (9). A study from China compared financial expenditures between families with and without children with ASD. The additional annual cost of raising a child with ASD was estimated at USD 2,984 (10). A study conducted in Egypt found that families with a child with ASD incur supplementary annual expenses ranging from USD 37,560 to USD 55,080 (11). Studies also indicate that treatment and education costs depend on whether a family has private or public insurance (12). In countries with well-developed social support systems and inclusive education, the costs associated with raising children with ASD are substantially lower. All monetary values were converted to USD using exchange rates as of January 1, 2025.

In Kazakhstan, where public funding for ASD-specific interventions is still developing, families often assume a considerable share of treatment-related costs, which may create financial challenges. Caregivers may experience employment disruptions, reduced household income, and increased stress, further compounding these challenges (13). These circumstances highlight the need for further policy development to reduce economic disparities, improve access to essential services, and support the well-being of caregivers and the quality of life of children with ASD and their families. Previous studies in Kazakhstan (14–17) have provided a general overview of ASD but have not examined the financial burden faced by families of children with ASD. Accordingly, this study aimed to assess financial expenditures and identify factors associated with financial difficulties among these families.

In this study, financial difficulties were defined as challenges experienced by parents of children with ASD due to insufficient financial resources to cover expenses related to healthcare, rehabilitation, educational services, and other ASD-related needs.

We assessed the presence and extent of financial difficulties faced by parents raising children with autism spectrum disorder (ASD) in Kazakhstan. We also analyzed differences between age groups and ethnic groups of children with ASD in order to provide a comprehensive overview of the current situation in the country. In addition, we assessed the influence of selected factors on the monthly expenditures associated with raising a child with ASD.

The findings of this study may provide valuable insights for policymakers and public health professionals seeking to improve the provision of financial and healthcare support for children with ASD.

## 2 Materials and methods

### 2.1 Study design and participants

This cross-sectional study examined financial difficulties among families of children with ASD. Contact information for eligible participants was obtained from the registries of the Regional Children’s Psychiatric Dispensary in the Karaganda region of Kazakhstan in 2023. The inclusion criteria were having a child under 18 years of age with a confirmed ASD diagnosis and providing parental consent to participate in the survey. All children had a clinical diagnosis of ASD established by a child psychiatrist in accordance with the International Classification of Diseases, Tenth Revision (ICD-10) criteria. Caregivers of these children were invited to participate. Families were excluded if the child did not have an officially confirmed ASD diagnosis or if the questionnaire contained substantial missing data that precluded statistical analysis.

The survey was administered both in person and online. Offline surveys were completed in paper form at the dispensary during medical appointments, while those who were unable to attend in person were invited to complete the survey online via Google Forms. Caregivers of these children completed a self-administered questionnaire. The questionnaire was available in Kazakh and Russian, and participants of all genders and ethnic backgrounds were eligible to participate. Each questionnaire was accompanied by an informed consent form. Of the 285 questionnaires distributed, 239 were eligible for analysis. Participants were recruited using convenience sampling. The sample size was determined by the number of caregivers of children with ASD who were available during the study period and agreed to participate. Data collection and processing were approved by the Ethics Committee of Karaganda Medical University (No. 71, dated May 19, 2021).

### 2.2 Questionnaire adaptation and description

**Figure 1:**
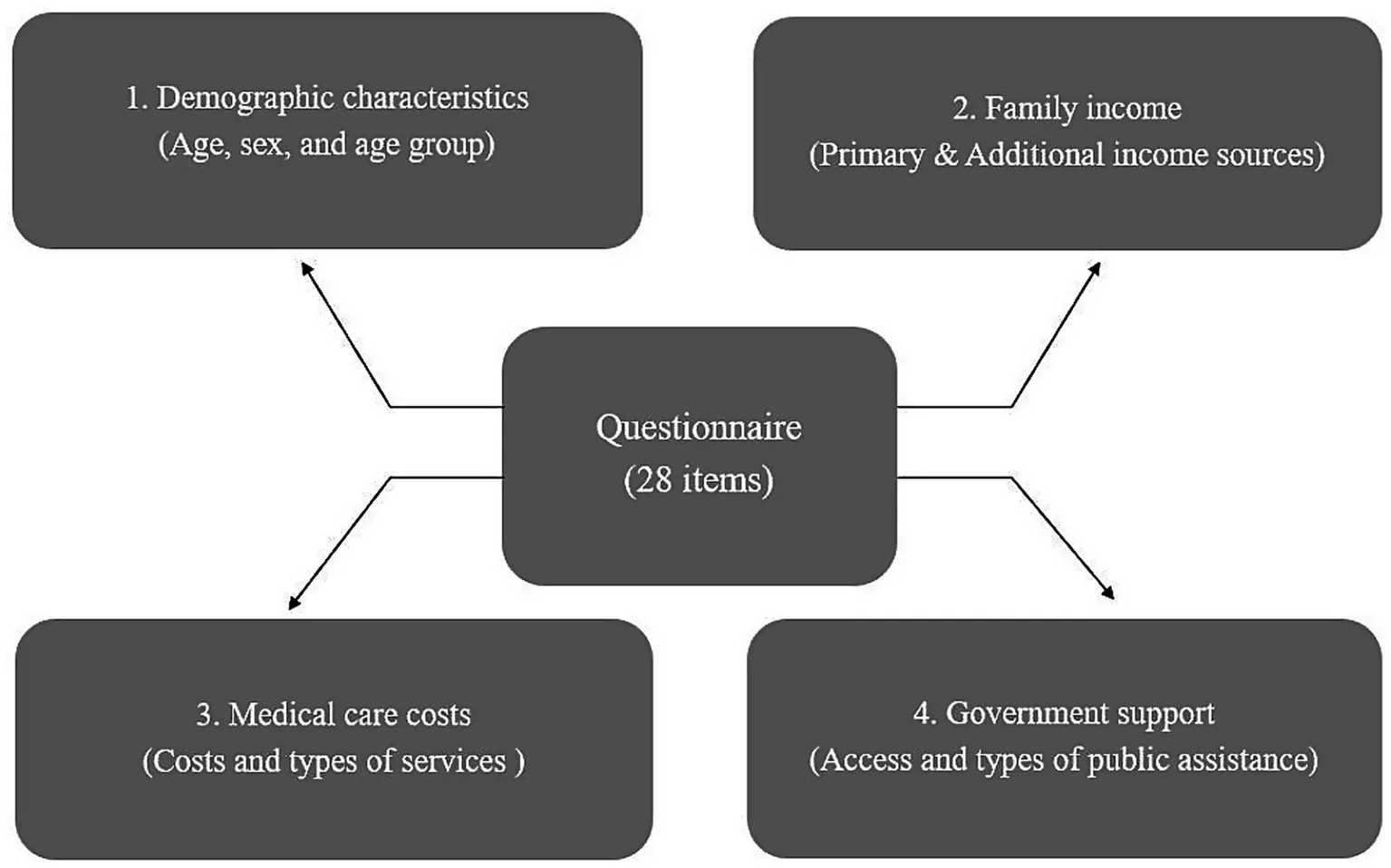
Structure of the study questionnaire.

To assess the socioeconomic burden associated with raising a child with ASD, we used the questionnaire entitled “Assessment of the Socioeconomic Costs of Families Raising a Child with ASD.” The questionnaire was adapted from an Australian study (9). The necessary intellectual property rights (Assessment of the Socioeconomic Costs of Families Raising a Child Diagnosed with ASD (Questionnaire), No. 1768, dated May 17, 2021) were obtained. The questionnaire was translated to Russian and Kazakh languages, then back-translated to English, and reconciled by a bilingual Russian-English and Kazakh-English psychiatrist and researcher. Formal validation and reliability testing of the adapted version were not performed. Before data collection, the questionnaire was pilot-tested to assess clarity, comprehensibility and feasibility. Minor wording revisions were made based on participants’ feedback (Figure 1).

### 2.3 Statistical analysis

The primary outcome of this study was self-reported financial difficulties among families raising a child with ASD. Financial difficulties were assessed using a single study-specific question asking caregivers whether they experienced financial hardship related to caring for their child with ASD. This binary variable (yes/no) served as the dependent variable in the logistic regression analyses. This study-specific measure was developed for the present study and was not based on a previously validated instrument.

A second outcome variable was the monthly family expenditures related to caring for a child with ASD (measured in Kazakhstani tenge), which served as the dependent variable in the multiple linear regression analysis. Data were analyzed using RStudio (version 2024.12.1 + 5,033; Posit PBC, Vienna, Austria). Categorical variables are presented as frequencies and percentages.

Comparisons of categorical variables were performed using Pearson’s chi-square test, with statistical significance set at *p* < 0.05. Multiple linear regression analysis was performed to identify factors associated with monthly family expenditures related to ASD care. Candidate independent variables included child characteristics (age, ethnicity, presence of comorbidities, and other health problems), family characteristics (marital status, number of children, family history of mental disorders, household income), receipt of government financial support, use of government intervention services, caregiver-reported social support (family and friends), and other sociodemographic variables. Variables were selected using a bidirectional stepwise procedure based on the Akaike Information Criterion (AIC). Regression coefficients (b) are reported as unstandardized estimates. Multivariable logistic regression analysis was performed to identify factors associated with the presence of financial difficulties. The dependent variable was the presence of financial difficulties (yes/no). Independent variables considered for inclusion were sociodemographic characteristics of the child and caregiver (child age, sex, ethnicity, caregiver age, marital status, place of residence), household characteristics (income, number of children, housing conditions), ASD-related expenditures, government financial support, healthcare utilization, satisfaction with healthcare services, perceived effectiveness of therapeutic interventions, and social support. Variable selection was also performed using bidirectional stepwise regression based on AIC. Results are presented as adjusted odds ratios (ORs) with corresponding *p*-values.

Before interpreting the regression models, model assumptions were evaluated. For the multiple linear regression model, multicollinearity was assessed using generalized variance inflation factors (GVIF), with all adjusted GVIF values (GVIF^(1/(2 × Df))) below 3, indicating no evidence of problematic multicollinearity. Homoscedasticity was assessed using the studentized Breusch–Pagan test, which showed no evidence of heteroscedasticity (BP = 53.43, *p* = 0.610). For the multivariable logistic regression models, multicollinearity was also assessed using GVIF, with all adjusted GVIF values below 3. Model calibration was evaluated using the Hosmer–Lemeshow goodness-of-fit test, which demonstrated good agreement between observed and predicted outcomes (χ^2^ = 3.64, df = 8, *p* = 0.888), indicating adequate model fit.

## 3 Results

A total of 239 questionnaires completed by caregivers of children with ASD were included in the analysis. Sociodemographic characteristics of the sample are presented in Table 1. Most children were male (79.08%). Most participants were of Kazakh ethnicity (76.98%), while 23.01% were of Russian or other ethnicities. The majority of respondents (90.79%) lived in urban areas, while 9.21% resided in rural areas. The mean age of the children was 8.56 years.

**Table 1:** Sociodemographic characteristics of the sample.

Variable	*n*	%
Gender (male/female)	189/50	79.08/20.92
Ethnicity (Kazakh/Russian/other)	184/48/7	76.98/20.08/2.93
Living area (urban/rural)	217/22	90.79/9.21
Marital status (married/divorced/other)	207/24/8	86.61/10.04/3.35
Number of children in the family (1, 2, ≥3)	184/38/17	76.99/15.89/7.11
Child’s age (mean ± SD)	8.56/3.25	

SD, standard deviation.

**Figure 2:**
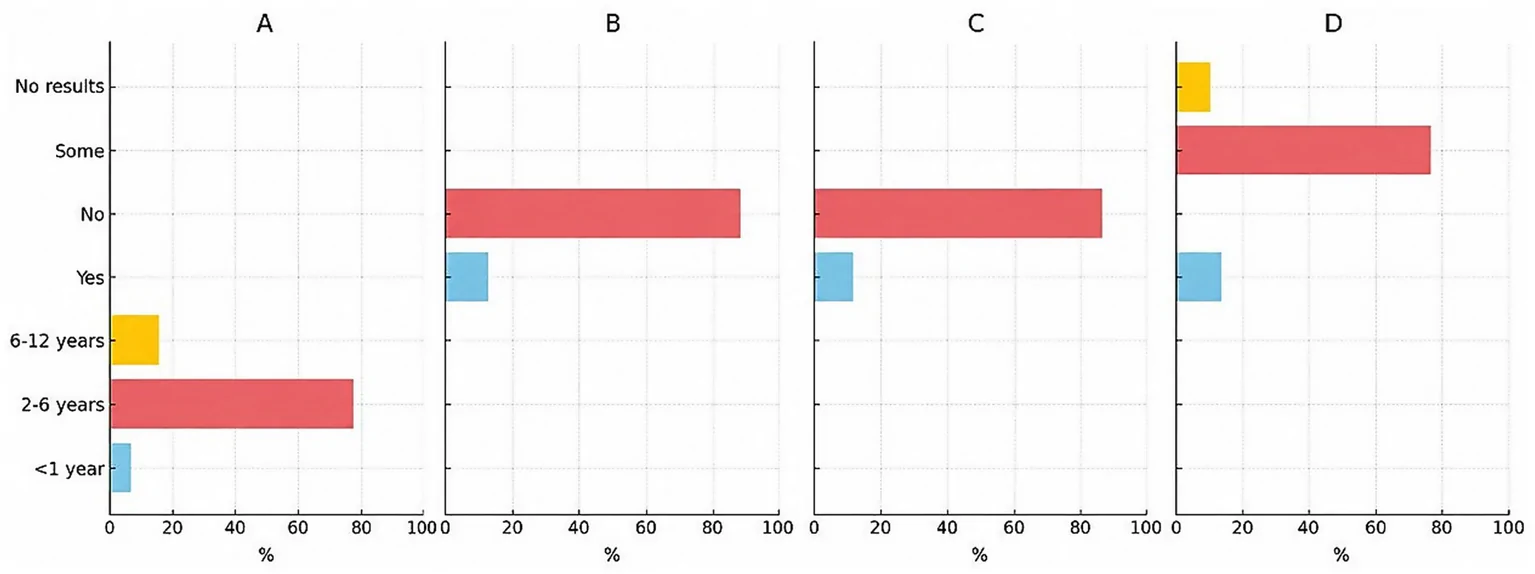
Distribution of responses regarding **(A)** age at ASD diagnosis, **(B)** comorbidities, **(C)** other health problems, and **(D)** perceived effectiveness of behavioral therapy.

Figure 2 presents a comparative analysis of key characteristics related to ASD diagnosis and associated factors in children. The first chart shows the distribution of children according to the age at which their diagnosis was made: most children (77.41%) were diagnosed between 2 and 6 years, while smaller proportions were diagnosed before 1 year (6.69%) or after 6 years (15.89%). The second and third charts depict the prevalence of comorbidities and other health conditions, with most children having no additional diagnoses (87.45 and 88.28%, respectively). The final chart shows caregivers’ reports of the perceived effectiveness of behavioral therapy, revealing that 77.41% of children showed improvement, while 15.89% had some progress, and 6.69% saw no results.

The financial challenges faced by families of children with ASD are summarized in Table 2. Most families (90.79%) reported experiencing financial difficulties related to caring for a child with ASD. Among respondents, 30.13% had to reduce their working hours, and 21.76% of parents left their jobs due to caregiving responsibilities. Non-medical costs accounted for the largest share of monthly expenditures (38.08%). A majority of families (64.02%) reported that they did not receive government financial support, whereas support from family members was reported in 80.75% of cases, and support from friends was limited (5.02%).

**Table 2:** Financial challenges and economic impact on families.

Variable	*n*	%
Reduction in working hours due to caring for a child with ASD (yes, no)	72/167	30.13/69.87
Employment termination due to caring for a child with ASD (yes, no)	52/187	21.76/78.24
Financial difficulties in the family due to ASD (yes, no)	217/22	90.79/9.21
Home modification costs (<200,000 KZT; 200,000–500,000 KZT; >500,000 KZT)	42/170/27	17.57/71.13/11.29
Largest categories of family expenditures (medication, medical services, non-medical expenses)	63/85/91	26.36/35.56/38.08
Government financial support (yes, no)	86/153	35.98/64.02
Support from friends (yes, no)	12/227	5.02/94.98
Support from family (yes, no)	193/46	80.75/19.25
Family income (<200,000 KZT; >200,000 KZT)	173/42	72.38/17.57

Table 3 presents the factors significantly associated with monthly family expenditures related to caring for a child with ASD. Families of children belonging to other ethnic groups had lower monthly expenditures (b = −138,293, *p* < 0.01). In contrast, the child’s health problems, number of children in the family, receipt of government financial support, attendance at state intervention centers, and support from family and friends were all significantly associated with higher monthly expenses. The highest average expenditures were observed in families whose children attended intervention centers (b = 260,605, *p* < 0.001).

**Table 3:** Multiple linear regression analysis of factors associated with monthly family expenditures.

Variable	*b*	*t*-value	*p*-value	95% CI (lower/upper)
Child’s ethnicity (other)	−138,293	−2.73	<0.01	−237826.81/243904.86
Presence of health problems in the child	75,477	2.73	<0.01	20452.15/130502.14
Number of children in the family	24,760	2.23	<0.05	2835.83/46683.27
Government financial support	64,824	3.33	<0.001	26497.54/103160.30
Attendance at state intervention centers	260,605	4.39	<0.001	143719.97/377489.77
Support from family	64,657	2.87	<0.01	20348.50/108965.41
Support from friends	100,977	2.78	<0.001	29555.44/172398.53
Intercept	108,980			
Adjusted *R*^2^	0.65			

Key sociodemographic factors associated with financial difficulties are presented in Table 4. Each additional year of the child’s age was associated with a 24% reduction in the odds of financial problems (OR = 0.76, *p* < 0.001), suggesting that younger age may be associated with greater financial strain. Families residing in urban areas had higher odds of reporting financial difficulties than those in rural areas (OR = 6.30, *p* < 0.05). Furthermore, married caregivers had 22% lower odds of facing financial difficulties (OR = 0.78, *p* < 0.05).

**Table 4:** Multivariable logistic regression analysis of sociodemographic factors associated with financial difficulties.

Variable	Odds ratio (95% CI)	*p*-value
Child’s age	0.76 (0.66–0.87)	<0.001
Urban area	6.30 (1.42–27.95)	<0.05
Married caregivers	0.78 (0.63–0.96)	<0.05

Table 5 presents additional determinants of families’ financial difficulties, with odds ratios indicating the strength and direction of these associations. Satisfaction with health services was associated with a lower likelihood of financial problems (OR = 0.14, *p* < 0.05). In contrast, families reporting some positive outcomes from therapeutic interventions were 8.90 times more likely to experience financial difficulties (OR = 8.90, *p* < 0.05). Receipt of government financial support was linked to lower odds of experiencing financial difficulties (OR = 0.09, *p* < 0.01).

**Table 5:** Multivariable logistic regression analysis of healthcare and support-related factors associated with financial difficulties.

Variable	Odds ratio (95% CI)	*p*-value
Satisfaction with health services	0.14 (0.02–0.63)	<0.05
Therapeutic interventions reported as providing some improvement	8.90 (1.08–76.23)	<0.05
Government financial support	0.09 (0.02–0.51)	<0.01

## 4 Discussion

This study is the first to examine factors associated with the financial burden experienced by families of children with ASD in Kazakhstan. Our findings indicate that raising a child with ASD places a substantial financial burden on families. The highest expenses were reported by families whose children with ASD attended intervention centers, who were younger, and who resided in urban areas. These results are consistent with international research (18–22).

Families in this study reported the greatest expenditures on home modifications and attendance at intervention centers. These findings underscore the ongoing need to create supportive environments for the development and rehabilitation of children with ASD, consistent with prior studies (23). Most caregivers reported receiving limited government financial support, indicating that families often play a central role in managing the financial, psychological, and physical demands of care (24–28). Nevertheless, substantial support from close relatives was reported, whereas assistance from non-relatives remained limited, aligning with earlier findings (29). This pattern may reflect strong intra-family ties and high levels of trust within Kazakh families.

Our study found that families of younger children with ASD incur higher expenses than those with older children, with a 24% reduction in costs for each additional year of age. Comparable findings from international research indicate that in the UK, expenses increase with age, whereas in the US, costs are higher during early childhood, reflecting the need for intensive therapy and care at younger ages (30). These patterns align with our results.

Our findings suggest that families residing in urban areas experienced greater financial burdens than those living in rural areas. This association may be explained by better access to diagnostic and therapeutic services in urban settings, which could result in higher out-of-pocket expenditures. However, this finding should be interpreted with caution, as it may also reflect unmeasured factors influencing the observed association, including differences in socioeconomic status, service availability, and healthcare-seeking behavior between urban and rural populations. Therefore, the observed association should not be interpreted as evidence of a causal relationship between place of residence and financial burden (31). However, Wang (22) reported the opposite pattern, with rural families bearing a larger share of expenses compared to urban families.

These findings highlight the substantial financial challenges faced by families of children with ASD and may inform governmental policy aimed at supporting these families (32). Public assistance is essential not only to alleviate economic burdens but also to improve the overall quality of life for children with ASD and their caregivers (33).

In Kazakhstan, services for children with ASD are delivered through the healthcare, education, and social protection sectors. Families may access educational and rehabilitation services, as well as monthly disability benefits. However, limited coordination among these sectors has resulted in the lack of comprehensive and reliable statistical data (34–37).

In our study population, the findings suggest that existing support measures may not fully offset the costs associated with raising children with ASD. Consequently, many families continue to incur substantial out-of-pocket expenses, particularly for behavioral and rehabilitative interventions.

International evidence indicates that coordinated healthcare, educational, and social support systems, together with early intervention and inclusive education policies, can improve access to services and reduce the burden experienced by families raising children with ASD (38–42). These approaches may provide valuable guidance for the further development of ASD-related policies in Kazakhstan.

This study has several limitations. First, because the study was conducted in a single region of Kazakhstan, the findings may not be representative of families of children with ASD throughout the country, which may limit the generalizability of the results. Nevertheless, the study provides valuable insights into the financial challenges experienced by these families in Kazakhstan. Second, participants were recruited from a single regional psychiatric dispensary using convenience sampling, which may have introduced selection bias. Third, because more than 90% of participants resided in urban areas, the findings may not be fully generalizable to rural populations. Differences in service availability, socioeconomic conditions, healthcare access, and healthcare-seeking behavior between urban and rural populations may have influenced the observed associations. Future studies that take these factors into account are needed to improve the representativeness of the findings and enable a more comprehensive assessment of the costs incurred by families raising children with ASD.

## 5 Conclusion

This study provides an overview of the financial challenges faced by families of children with ASD in Kazakhstan. The findings underscore the need for comprehensive government support, which could improve the quality of life for these families, as the financial burdens associated with raising a child with ASD often persist throughout life.

## Data Availability

The original contributions presented in the study are included in the article/supplementary material, further inquiries can be directed to the corresponding author.
